# ‘*Mycoplasma hominis* does not share common risk factors with other genital pathogens’: Findings from a South African pregnant cohort

**DOI:** 10.4102/sajid.v36i1.207

**Published:** 2021-05-19

**Authors:** Meleshni Naicker, Fazana Dessai, Ravesh Singh, Nireshni Mitchev, Partson Tinarwo, Nathlee S. Abbai

**Affiliations:** 1 School of Clinical Medicine Laboratory, College of Health Sciences, University of KwaZulu-Natal, Durban, South Africa; 2 Department of Medical Microbiology, College of Health Sciences, University of KwaZulu-Natal, Durban, South Africa; 3 Department of Biostatistics, Faculty of Health Sciences, University of KwaZulu-Natal, Durban, South Africa; 4 Department of Clinical Medicine Laboratory, Faculty of Health Sciences, University of KwaZulu-Natal, Durban, South Africa

**Keywords:** *Mycoplasma hominis*, vaginal infections, pregnant women, risk factors, HIV infection, bacterial vaginosis

## Abstract

**Background:**

The role of *Mycoplasma hominis* (*M. hominis*) as a genital tract pathogen was still debatable. This study identified the risk factors associated with the prevalence of *M. hominis* in South African pregnant women.

**Methods:**

This was a cross-sectional analysis of *n* = 221 prenatal patients attending a Durban hospital during November 2017 to April 2018. *M. hominis* was detected from urine samples using the quantitative polymerase chain reaction. The population characteristics were described using frequencies stratified by the infection status of *M. hominis.* In addition, a univariate analysis was used to assess the relationship between each risk factor and infection status. The analysis further considered logistic regression to assess the influence of these risk factors univariately and in the presence of other factors. The coinfection rate between *M. hominis* and bacterial vaginosis (BV), *Trichomonas vaginalis* (*T. vaginalis*), *Mycoplasma genitalium (M. genitalium)* and *Candida* species was also determined. All the tests were conducted at 5% level of significance.

**Results:**

The prevalence of *M. hominis* in this study population was 48% (106/221). In the univariate analysis, factors significantly associated with *M. hominis* positivity included having past abnormal vaginal discharge (*p* = 0.037), having current abnormal vaginal discharge (*p* = 0.010) and a borderline significance (*p* = 0.052), which were noted for previous pre-term delivery. However, none of these factors were sustained in the multivariate analysis. There was a statistically significant association between *M. hominis* and BV positivity (*p* < 0.001). Similarly, *M. hominis* and *M. genitalium* positivity was significant (*p* = 0.006).

**Conclusion:**

This study showed that *M. hominis* does not share common risk factors with known genital tract pathogens in a population of pregnant women and therefore cannot be considered a genital tract pathogen.

## Introduction

According to the World Health Organization (WHO), there are more than 376 million incidences of sexually transmitted infections (STIs) reported worldwide annually, thus making these infections an important public health concern.^1^ Depending on the causative agent, an STI is usually manifested in a skin lesion, secretion, vaginal discharge, wart or blister.^2^ The vaginal microbiome consists of numerous microorganisms, including members of the class Mollicutes, order Mycoplasmatales (mycoplasmas and ureaplasmas).^3^

Mollicutes are included in STIs, but they are also found in healthy individuals.^2^ The most important Mollicutes colonising the female genital tract are *Ureaplasma urealyticum, Ureaplasma parvum, Mycoplasma hominis* (*M. hominis*) and *Mycoplasma genitalium* (*M. genitalium*)^4^ Genital mycoplasmas colonise the vaginal tract of up to 80% of pregnant and non-pregnant women.^5^

*Mycoplasma hominis* is considered to be an important opportunistic pathogen implicated in urogenital infections and complicated pregnancy outcomes.^6^ These include pelvic inflammatory disease, endometritis, chorioamnionitis and postpartum fever, resulting in complications such as infertility, spontaneous abortion, stillbirth, preterm birth, low birth weight and perinatal mortality.^7,8,9,10,11,12^

An earlier study had provided evidence stating that *M. hominis* is not a vaginal pathogen in adults.^13^ However, a study published in the same year by Arya et al. referred to *M. hominis* as a vaginal pathogen because of its association with *Trichomonas vaginalis* (*T. vaginalis*).^14^ There have been limited studies since 2001, which have contributed to resolving the discordance regarding the role of *M. hominis* as a genital tract pathogen. The aim of the current study was to describe the prevalence and factors associated with *M. hominis* in pregnant women as well as its likelihood of being considered as a genital tract pathogen.

## Materials and methods

### Study setting and population

This study was a sub-study of a larger study that investigated the laboratory-based diagnosis of vaginal infections in pregnant women from Durban. The larger study included *n* = 273 women, 18 years and older from all gestational ages who are willing to provide written informed consent. The study population was recruited from the King Edward VIII hospital in Durban, KwaZulu-Natal between October 2017 and April 2018. All patients were outpatients. There was a 10% refusal rate by the women during screening. A questionnaire was administered to collect data on the women’s demographics, sexual behaviour and clinical information. All interviews were conducted in private, and all study-related information was stored securely. All records and specimens had been identified by study identification numbers only to maintain participant confidentiality.

Only participants who had given written informed consent were included in the study. The study did not collect data on whether the women were experiencing any complications during pregnancy. During the study visit, women were asked to provide self-collected vaginal swab and urine samples. The women were tested for HIV at the clinic as part of routine care. Permission to obtain data on HIV status was obtained from the women. Because of the cross-sectional design of the study, the women were not followed up to collect information on pregnancy outcomes.

For the sub-study, *n* = 221 urine DNA extracts were available for analysis. Laboratory testing and analyses were performed at the School of Clinical Medicine Laboratory, University of KwaZulu-Natal.

### Study procedures

#### Data collection

At enrolment, a face-to-face questionnaire was administered to collect data on the women’s demographics (age, level of education and marital status), sexual behaviour (condom use, number of lifetime sex partners, age of sexual debut, partner having other partners, intravaginal practices, cohabitation status and recreational habits such as smoking and consuming alcohol) and clinical information (gestational age, history of previous pregnancies and history of STIs).

#### Detection of *Mycoplasma homin is* from urine

A sensitivity detection assay was performed on the urine DNA extracts. DNA extraction involved a starting volume of 10 ml of urine. A standardised starting volume was used across all samples. The urine was centrifuged for 45 min at 14 000 × g and the supernatant was discarded. Total DNA was then extracted from recovered sample pellets using the PureLink^TM^ Microbiome DNA Purification Kit (Thermo Fisher Scientific, United States) in accordance with the manufacturer’s instructions. The DNA concentration was measured using a NanoDrop Spectrophotometer (Thermo Fisher Scientific, United States). Resulting DNA concentrations ranged from 3.1 3 ng/µL to 75.3 ng/µL with A_260_/A_280_ ratios in the range of 0.80–1.91.

*Mycoplasma hominis* was detected using the TaqMan Real-time polymerase chain reaction (PCR) (sensitivity) assay (Thermo Fisher Scientific, United States) using commercially available primers and probes specific for *M. hominis* (Ba04646255_s1). The assays were run on the Quant Studio 5 Real-time PCR detection system (Thermo Fisher Scientific, USA).

Each PCR reaction was performed in a final volume of 5 uL comprising 0.25 uL of FAM-labelled probe/primer mix, 1.25 uL of Fast Start 4x probe master mix (Thermo Fisher, Part No. 4444434), 1.5 uL of template DNA and nuclease-free water.

Non-template and positive controls (TaqMan™ Vaginal Microbiota Extraction Control; cat no. A32039) were also included. Amplification was performed at 95° C for 30 s followed by 45 cycles comprising denaturation at 95° C for 3 s and annealing at 60° C for 30 s. Detection of amplified fluorescent products was carried out at the end of the annealing phase. The raw fluorescence data that included the C_T_ mean values were automatically generated using the Quant Studio 5 Real-time PCR system software.

#### Detection of *Mycoplasma genitalium* from urine

*Mycoplasma genitalium* was detected using the TaqMan Real-time PCR (sensitivity) assay (Thermo Fisher Scientific, United States) using commercially available primers and probes specific for *M. hominis* (Ba04646249_sl). The reaction and cycling conditions were as per the *M. hominis* assay conditions.

#### Detection of bacterial vaginosis, *Trichomonas vaginalis* and *Candida* species from vaginal swabs

The presence of BV, *T. vaginalis* and *Candida* species was detected using the BD Max^TM^ Vaginal Panel assay (Becton Dickinson, United States) from a single vaginal swab. The assay was performed as per the manufacturer’s recommendations.

#### Statistical data analyses

The statistical data analysis was conducted in a freely available Statistical Computing Environment, R software, version 3.6.3 using the RStudio platform. Initially, the population characteristics were described using frequencies stratified by the infection status of the pathogens.

In addition to the frequencies, univariate analysis was used to assess the relationship between each risk factor and the pathogen infection status. The available continuous variable had a skewed distribution calling for a non-parametric test involving a rank-sum test. On the other hand, the categorical risk factors were univariately assessed using the Chi-Square test or the Fisher’s exact test in the case of smaller expected frequencies. The significant risk factors were used to fit univariate logistic regressions in order to quantify their relationships with the outcome in terms of odds ratios (ORs). The analysis further considered multiple logistic regression to assess the influence of these univariately significant risk factors in the presence of the other factors. All the tests were conducted at 5% level of significance.

### Ethical considerations

Ethics approval for this study was granted by the Biomedical Research Ethics Committee (BREC) of the University of KwaZulu-Natal (BE214/17).

## Results

### Characteristics of the population according to *Mycoplasma hominis* status

The prevalence of *M. hominis* in the study population was 48% (106/221). Table 1 shows the factors in relation to *M. hominis* status. There was no significant association (*p* > 0.05) between the majority of demographic variables and the prevalence of *M. hominis*. Of the 106 *M. hominis* positive women, a small proportion of them, 20.8% (22/106) had attended college/university when compared to the majority of women (69.8% [74/106]) who attended high school. When considering the behavioural factors, it was shown that amongst the 106 *M. hominis*-positive women most women 83.0% (88/106) reported having a regular sex partner when compared to women not reported having a regular sex partner (17%). A majority of positive women engaged in their first sex at an early age of 15 and 20 years, 72.6% (77/106) followed by delayed sex at > 20 years of age constituting 21.7% (23/106). The dominant number of lifetime sex partners amongst the *M. hominis*-positive women was 2–4 partners 51.9% (55/106) compared to just one lifetime sex partner 22.6% (24/106) and > 4 lifetime sex partners 25.5% (27/106). The results also showed that 71.7% (76/106) of the *M. hominis*-positive women did not use a condom at their last sex and 90.6% (96/106) did not engage in intravaginal practices. Despite the high proportion of women reporting risky behavioural practices, there was no significant association (*p* > 0.05) between most of these potential risk factors and the prevalence of *M. hominis* (Table 1). With respect to the clinical symptoms, it was found that amongst the *M. hominis*-positive women, almost half did not experience past episodes of abnormal vaginal discharge 50.5% (53/106), which was a significantly (*p* = 0.037) smaller proportion when compared to 64.3% (74/115) who did not experience past episodes of abnormal vaginal discharge amongst the *M. hominis*-negative women. Similarly, at the time of enrolment into the study, a significantly (*p* = 0.010) smaller proportion 56.6% (60/106) of women did not experience current abnormal vaginal discharge amongst the *M. hominis*-positive women compared to 73% (84/115) within the *M. hominis*-negative women. For the women who tested *M. hominis* positive, a higher proportion 75.5% (80/106) of them did not experience a previous pre-term delivery. However, the association between pre-term delivery and *M. hominis* infection was at a threshold significance (*p* = 0.052) with more inclination towards insufficient evidence to suggest that the association between pre-term delivery and *M. hominis* infection indeed exists (Table 1).

**TABLE 1a:** Characteristics of the study population by *Mycoplasma hominis* status.

Variable	*M. hominis*		Overall (*n* = 221)	*p*-value
	Negative (*n* = 115)	Positive (*n* = 106)		
**Age**	**-**	**-**	**-**	**0.490**
Mean ± SD	28.6 ± 6.14	28.0 ± 5.95	28.3 ± 6.04	-
CV%	21.5	21.3	21.3	-
Median	28.0	27.0	27.0	-
Q1; Q3	24.0; 33.0	24.0; 33.0	24.0; 33.0	-
Min–Max	18.0–43.0	18.0–43.0	18.0–43.0	-

**TABLE 1b:** Characteristics of the study population by *Mycoplasma hominis* status.

Variable	*M. hominis*				Overall (*n* = 221)		*p*-value
	Negative (*n* = 115)		Positive (*n* = 106)		*n*	%	
	*N*	%	*N*	%		
**Current abnormal vaginal discharge**	-	-	-	-	-	-	0.01
No	84	73.0	60	56.6	144	65.2	-
Yes	31	27.0	46	43.4	77	34.8	-
**Symptoms of STIs in the past 3 months**	-	-	-	-	-	-	0.08
No	102	88.7	85	80.2	187	84.6	-
Yes	13	11.3	21	19.8	34	15.4	-
**Level of education**	-	-	-	-	-	-	0.061
Primary and below	5	04.3	10	09.4	15	06.8	-
High school	72	62.6	74	69.8	146	66.1	-
College/University	38	33.0	22	20.8	60	27.1	-
**Marital status**	-	-	-	-	-	-	0.191
No	94	82.5	94	88.7	188	85.5	-
Yes	20	17.5	12	11.3	32	14.5	-
**Has a regular sexual partner**	-	-	-	-	-	-	0.936
No	20	17.4	18	17.0	38	17.2	-
Yes	95	82.6	88	83.0	183	82.8	-
**Living with sexual partner**	-	-	-	-	-	-	-
No	69	60.0	67	63.2	136	61.5	-
Yes	46	40.0	39	36.8	85	38.5	-
**Age at first sex**	-	-	-	-	-	-	0.71
< 15	5	04.3	6	05.7	11	05.0	-
15–20	89	77.4	77	72.6	166	75.1	-
> 20	21	18.3	23	21.7	44	19.9	-
**Lifetime number of sexual partners**	-	-	-	-	-	-	0.171
1	37	32.2	24	22.6	61	27.6	-
4-Feb	58	50.4	55	51.9	113	51.1	-
> 4	20	17.4	27	25.5	47	21.3	-
**Partner has other partners**	-	-	-	-	-	-	0.116
No/Do not know	86	74.8	69	65.1	155	70.1	-
Yes	29	25.2	37	34.9	66	29.9	-
**Condom use**	-	-	-	-	-	-	0.47
Never	37	32.2	39	36.8	76	34.4	-
Always	78	67.8	67	63.2	145	65.6	-
**Condom use at last sexual act**	-	-	-	-	-	-	0.193
No	73	63.5	-	-	149	67.4	-
Yes	42	36.5	-	-	72	32.6	-
**Smokes**	-	-	-	-	-	-	0.317
No	112	97.4	-	-	212	95.9	-
Yes	3	02.6	-	-	9	04.1	-
**Consumes alcohol**	-	-	-	-	-	-	0.08
No	107	93.0	-	-	198	89.6	-
Yes	8	07.0	-	-	23	10.4	-
**Intravaginal practices**	-	-	-	-	-	-	0.973
No	104	90.4	-	-	200	90.5	-
Yes	11	09.6	-	-	21	09.5	-
**Trimester of pregnancy**	-	-	-	-	-	-	0.898
1st	10	08.7	-	-	21	09.5	-
2nd	40	34.8	-	-	75	33.9	-
3rd	65	56.5	-	-	125	56.6	-
**Previous pre-term delivery**	-	-	-	-	-	-	0.052
No	96	83.5	-	-	176	79.6	-
Yes	15	13.0	-	-	40	18.1	-
Missing	4	03.5	-	-	5	02.3	-
**Past miscarriage**	-	-	-	-	-	-	0.191
No	80	69.6	-	-	162	73.3	-
Yes	35	30.4	-	-	59	26.7	-
**Past spontaneous abortion**	-	-	-	-	-	-	0.259
No	107	93.0	-	-	201	91.0	-
Yes	8	07.0	-	-	20	09.0	-
**Previous abnormal vaginal discharge**	-	-	-	-	-	-	0.037
No	74	64.3	-	-	127	57.7	-
Yes	41	35.7	-	-	93	42.3	-
**Previously treated for STIs**	-	-	-	-	-	-	0.283
No	69	60.0	-	-	125	56.6	-
Yes	46	40.0	-	-	96	43.4	-

STIs, sexually transmitted infections.

### Risk factors associated with *Mycoplasma hominis* infection

Table 2 shows the risk factors associated with *M. hominis* infection for *p* < 0.1. In this univariable analysis, having a current abnormal discharge was two times more likely to test positive for *M. hominis* (OR: 2.08, 95% Confidence Interval [CI]: 1.19–3.67, *p* = 0.011). Furthermore, it was shown that having a previous abnormal vaginal discharge increased the risk of testing *M. hominis* positive by 77% (OR: 1.77, CI 1.03–3.05, *p* = 0.038). Finally, this univariate analysis demonstrated that having attained a college level of education reduced the women’s risk of being infected by 71% (OR: 0.29, 95% CI: 0.08–0.92, *p* = 0.042). By adjusting these factors amongst themselves, their relationships with prevalent *M. hominis* were found to be statistically insignificant (*p* > 0.05).

**TABLE 2:** Univariate and multiple regression analysis of risk factors associated with *Mycoplasma hominis* infection.

Explanatory	OR (Unadjusted)			OR (Adjusted)		
	OR	95% CI	*p*-value	OR	95% CI	*p*-value
Current abnormal vaginal discharge	2.08	1.19–3.67	0.011	1.58	0.77–3.28	0.211
No discharge (Referent)	1	-	-	1	-	-
STI symptoms	1.94	0.93–4.19	0.083	0.99	0.39–2.52	0.989
No STI symptoms (Referent)	1	-	-	1	-	-
Education (High school)	0.51	0.15–1.52	0.245	0.59	0.17–1.83	0.374
Education (College/University)	0.29	0.08–0.92	0.042	0.33	0.09–1.11	0.080
Education (Primary school) (Referent)	1	-	-	1	-	-
Consumes alcohol	2.2	0.91–5.70	0.086	1.97	0.76–5.54	0.175
Does not consume alcohol (Referent)	1	-	-	1	-	-
Had a previous pre-term baby	2	1.00–4.13	0.054	1.61	0.77–3.45	0.207
No previous pre-term delivery (Referent)	1	-	-	1	-	-
Past abnormal vaginal discharge	1.77	1.03–3.05	0.038	1.44	0.79–2.62	0.231
No past discharge (Referent)	1	-	-	1	-	-

STI, sexually transmitted infection.

### Coinfection between *Mycoplasma hominis* bacterial vaginosis, *Trichomonas vaginalis, Candida* species and *Mycoplasma genitalium*

There was a statistically significant association between *M. hominis* and BV positivity (*p* < 0.001) (Table 3). That is, amongst the 106 women who tested positive for *M. hominis*, 66.0% also tested positive for BV and this was a significantly higher proportion when compared to 27.8% (32/115) BV positives amongst the *M. hominis*-negative women. Similarly, there was a significant association between *M. hominis* and *M. genitalium* positivity (*p* = 0.006). The coinfection rate between *M. hominis* and *M. genitalium* was 4.98% (11/221) constituting 10.4% (11/106) of the *M. hominis*-positive women (Table 3). Despite high co-infection rates between *M. hominis* and *T. vaginalis* (14.2% of the *M. hominis* positive) and *M. hominis* and *Candida* species (59.4% of the *M. hominis* positive), these associations were not significant (*p* = 0.130 and *p* = 0.853, respectively) (Table 3).

**TABLE 3:** Coinfection between *Mycoplasma hominis* and genital tract infections.

*M. hominis*	Negative (*N* = 115)		Positive (*N* = 106)		Overall (*N* = 221)		*p*-value
	*n*	%	*N*	%	*N*	%	
Bacterial vaginosis	-	-	-	-	-	-	< 0.001
Negative	74	64.3	29	27.4	103	46.6	-
Positive	32	27.8	70	66.0	102	46.2	-
Missing	9	7.8	7	6.6	16	7.2	-
*Candida* species	-	-	-	-	-	-	0.853
Negative	47	40.9	42	39.6	89	40.3	-
Positive	67	58.3	63	59.4	130	58.8	-
Missing	1	0.9	1	0.9	2	0.9	-
*Trichomonas vaginalis*	-	-	-	-	-	-	0.130
Negative	105	91.3	90	84.9	195	88.2	-
Positive	9	7.8	15	14.2	24	10.9	-
Missing	1	0.9	1	0.9	2	0.9	-
*Mycoplasma genitalium*	-	-	-	-	-	-	0.006
Negative	113	98.3	95	89.6	208	94.1	-
Positive	2	1.7	11	10.4	13	5.9	-

### Predicting the risk of *Mycoplasma hominis* infection in the presence of other genital infections

The results in Table 4 showed that having a prevalent BV infection significantly increased the risk of acquiring *M. hominis* by 5-fold in both the unadjusted (OR: 5.19, 95% CI: 2.75–10.10, *p* < 0.001) and adjusted analyses (OR: 5.19, 95% CI: 2.75–10.10, *p* < 0.001). The results further revealed that being *M. genitalium* positive doubled the chances of *M. hominis* infection as compared to having been BV positive. *M. genitalium*-positive women had an increased risk of *M. hominis* infection by 12-fold (OR: 12.28, 95% CI: 2.28–227.76, *p* = 0.018) and 10-fold (OR: 9.54, 95% CI: 1.58–185.73, *p* = 0.041) in the unadjusted and adjusted analyses, respectively. However, the stepwise regression suggested that *Candida* species and *T. vaginalis* were not important in predicting the likelihood of *M. hominis* infection. That is, without taking *Candida* species and *T. vaginalis* into consideration, the refined results still show that BV increased the risk for *M. hominis* infection by close to five-fold (OR: 4.87, 95% CI: 2.61–9.31, *p* < 0.001) and *M. genitalium* increased the risk for *M. hominis* infection by close to nine-fold (OR: 8.90, 95% CI: 1.52–170.55, *p* = 0.045).

**TABLE 4:** Risk of acquiring *Mycoplasma hominis* in the presence of other genital infections.

Variable	Unadjusted			Adjusted			Stepwise		
	OR	CI	*p*-value	OR	CI	*p*-value	OR	CI	*p*-value
BV positive	5.24	2.84–9.92	< 0.001	5.19	2.75–10.10	< 0.001	4.9	2.61–9.31	< 0.001
BV negative (Referent)	1	-	-	1	-	-	-	-	-
*Candida* species positive	0.93	0.52–1.67	0.805	1.37	0.71–2.71	0.351	-	-	-
*Candida* species negative (Referent)	1	-	-	1	-	-	-	-	-
*T. vaginalis* positive	1.69	0.69–4.31	0.254	1.99	0.73–5.59	0.181	-	-	-
*T. vaginalis* negative (Referent)	1	-	-	1	-	-	-	-	-
*M. genitalium* positive	12.3	2.28–227.76	0.018	9.54	1.58–185.73	0.041	8.9	1.52–170.55	0.045
*M. genitalium* negative (Referent)	1	-	-	1	-	-	-	-	-

BV, bacterial vaginosis; *T., Trichomonas; M., Mycoplasma.*

### Predicting the risk of bacterial vaginosis infection in the presence of other genital infections

Table 5 shows that a woman who is *M. hominis* positive had an increased risk of BV infection by 5-fold both univariately (OR: 5.37, 95% CI: 2.96–9.98, *p* < 0.001) and by controlling for the other genital infections (OR: 5.44, 95% CI: 2.94–10.39, *p* < 0.001). This confirms that the odds of *M. hominis* infection given BV infection or vice versa are the same (5-fold). Although *Candida* species infection status was not significantly associated with BV infection, the results showed that it is important to gather data on *Candida* species alongside that of *M. hominis* in order to have a better prediction of the BV infection. Unlike for *M. hominis, M. genitalium* was found to have no leads on the BV infection. However, the *T. vaginalis* infection status could not indicate the likelihood of infection also for either *M. hominis* or BV.

**TABLE 5:** Risk of acquiring bacterial vaginosis in the presence of other genital infections.

Variable	Unadjusted			Adjusted			Stepwise		
	OR	CI	*p*-value	OR	CI	*p*-value	OR	CI	*p*-value
*Candida* species positive	0.64	0.36–1.13	0.127	0.62	0.33–1.16	0.139	0.62	0.33–1.15	0.128
*Candida* species negative (Referent)	1	-	-	1	-	-	-	-	-
*T. vaginalis* positive	0.99	0.42–2.34	0.979	0.70	0.27–1.81	0.459	-	-	-
*T. vaginalis* negative (Referent)	1	-	-	1	-	-	-	-	-
*M. hominis* positive	5.37	2.96–9.98	< 0.001	5.44	2.94–10.39	< 0.001	5.43	2.98–10.15	< 0.001
*M. hominis* negative (Referent)	1	-	-	1	-	-	-	-	-
*M. genitalium* positive	3.16	0.91–14.60	0.091	1.32	0.34–6.49	0.703	-	-	-
*M. genitalium* negative (Referent)	1	-	-	1	-	-	-	-	-

*T., Trichomonas; M., Mycoplasma.*

### Association between *Mycoplasma hominis* and HIV infection

**FIGURE 1:**
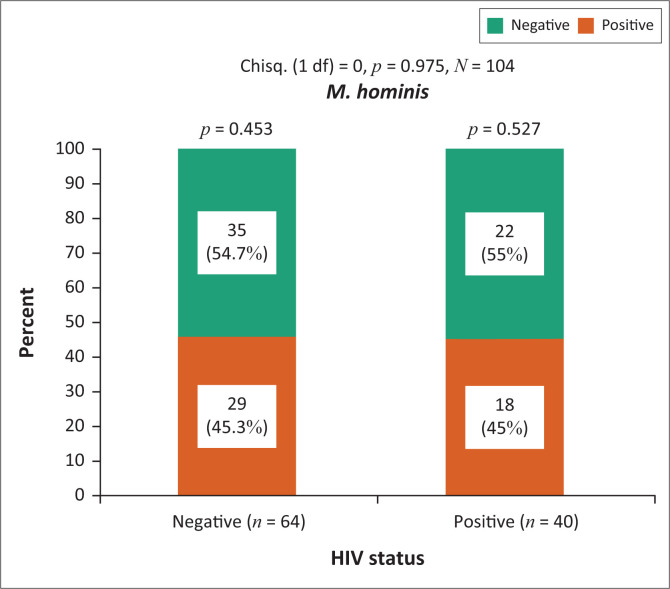
Prevalence of *Mycoplasma hominis* amongst HIV infected and uninfected women.

For this analysis, the data on HIV status were available for *n* = 104 women. A large proportion of the women refused to provide these data. For the 104 women for whom data were available, it was shown that the prevalence of coinfection between HIV and *M. hominis* was 17.3% (11/104), constituting 45% (18/40) of the *M. hominis*-positive women. However, there was no statistically significant association between prevalent HIV and prevalent *M. hominis* (*p* = 0.975) (Figure 1). That is, the prevalence of *M. hominis* infection amongst the HIV-negative and HIV-positive women was similar at approximately 45%.

## Discussion

To the best of our knowledge, this is the first study to provide an estimate on the prevalence of *M. hominis* in pregnant women from the Durban area in South Africa. We report a prevalence estimate of 48% for *M. hominis* in this study population. Our data are consistent with a previous study conducted in South Africa where Redelinghuys and colleagues also reported high prevalence data for *M. hominis* (50.7%) in pregnant women from Gauteng, South Africa.^5^

Going back to the overall study aim, which was to identify risk factors associated with *M. hominis* as well as to determine if *M. hominis* shared risk factors with other genital infections, the following factors were significantly associated with the prevalence of *M. hominis:* level of education, current abnormal vaginal discharge, past abnormal vaginal discharge and past pre-term delivery. A high proportion of women in this study had attained a high school level of education. There was a borderline significance between this variable and *M. hominis* status in this study. Previous studies conducted in women from KwaZulu-Natal, South Africa, have shown that women with a lower level of schooling, that is, less than high school, have more prevalent genital infections.^15,16,17^ Abbai et al. showed that women with a lower level of education are at high risk of having multiple STIs (*p* = 0.034).^15^ Similarly, Naidoo et al. also showed that women with the prevalent STIs reported less than high school education (*p* < 0.0001).^16^ A significant association between low level of education and prevalence of the viral STI, Herpes simplex virus-2 was also found (*p* = 0.021).^17^

In this study, current abnormal vaginal discharge was significantly associated with the prevalence of *M. hominis*. The majority of women who tested positive did not report symptoms of discharge. A similar observation was reported by Dessai et al. for a population of pregnant women from Durban, where the majority of women who tested positive for *Candida* reported not having a current abnormal vaginal discharge (*p* < 0.001).^18^ In another recent study conducted by Mabaso et al., it was shown that the majority of women who tested positive for *T. vaginalis* did not report current symptoms of abnormal vaginal discharge (*p* = 0.011).^19^ In this study, past abnormal vaginal discharge was also associated with the prevalence of *M. hominis*. The study conducted by Dessai et al. showed a borderline significance (*p* = 0.06) for past discharge and prevalent *T. vaginalis* infections.^18^ Previous studies have shown an association between pre-term deliveries and *M. hominis* infection.^7,12^ However, the present study did not show a positive association between past history of pre-term delivery and the prevalence of *M. hominis.* An explanation for this could be because of the small overall number of women (*n* = 40) who reported this event. For future association studies, a larger number of women reporting this event may be needed to see a positive association.

In the current study, a univariate and multivariate analysis was performed in order to determine if the significant variables described thus far were truly risk factors associated with *M. hominis*. Reported symptoms of abnormal vaginal discharge were shown to be significantly associated (*p* < 0.05) with prevalent *M. hominis* in the univariate analysis. Women who presented with a current or previous abnormal discharge were two times and 77% more likely to develop prevalent *M. hominis*. These findings are consistent with another study that reported on the prevalence of genital mycoplasma species, especially *M. hominis,* in patients presenting with vaginal discharge.^20^ However, this association was not sustained in the multivariable analysis, indicating that the abnormal vaginal discharge may not be a true risk factor for acquiring *M. hominis*. As shown in this study, there was a high coinfection rate of *M. hominis* with other infections such as BV, *T. vaginalis, M. genitalium* and *Candida* species. These coinfections could have contributed to the discharge and not necessarily *M. hominis*.

This is not in keeping with another published study conducted in pregnant women, which showed the association of abnormal vaginal discharge as a true risk factor for another genital pathogen.^19^

The current study also showed that obtaining a tertiary level of education had significantly reduced the women’s risk of infection by 71%. This finding is consistent with other previous studies conducted in KwaZulu-Natal.^16,17^ Abbai and co-workers reported a significant association between HSV-2 infection and women who had received a lower level of education.^17^ Similarly, Naidoo et al. associated women receiving lower level education with an increased risk of having a prevalent STI.^16^ However, unlike the findings described by Abbai et al.^17^ and Naidoo et al.,^16^ level of education was not significant in the multivariable analysis. Therefore, level of education cannot be deemed as a true low risk factor in this study.

In this study, high coinfection rates were observed for *M. hominis* with BV, *Candida, T. vaginalis, M. genitalium* and HIV. However, only coinfection rates between *M. hominis* and BV and *M. hominis* and *M. genitalium* were shown to be significant. In the adjusted analysis, BV was shown to significantly increase the risk for *M. hominis* by five-fold. A study conducted by Sanchez-Garcia et al. showed that BV was also significantly associated with an increased risk of positivity for *M. hominis* (OR: 25.9, 95 % CI: 7.2–93.0; *p* = 0.001).^21^

According to Panos, the presence of *M. genitalium* infection was associated with the presence of Mycoplasmataceae family members such as *M. hominis* and *Ureaplasma* species, more particularly, *Ureaplasma* species.^22^ Testing for the presence of *Ureaplasma* species in the study cohort is a future research direction.

This study was limited in that samples were collected from pregnant women attending a single antenatal facility. However, the hospital from which the women were sampled in this study serves as a central hospital for women from around the Durban area, thereby making the population more generalised. A second limitation is the lack of data on pregnancy outcomes in relation to the prevalent infections. Because of the cross-sectional nature of this study, the data were not collected; however, this limitation will be addressed in future studies. Finally, a full dataset on HIV status was not available for this study, because of the refusal to provide the data by the study women and therefore, this study was unable to draw sound conclusions regarding the association between *M. hominis* and HIV infections.

## Conclusion

To date, there remains uncertainty regarding the role of *M. hominis* as a genital tract pathogen. The current study has now provided evidence from a South African-based pregnant population, indicating that *M. hominis* does not share common predisposing risk factors with that of known genital tract pathogens as well as the causative agents of STIs. Based on these study findings, *M. hominis* cannot be considered a genital tract pathogen. Previous studies have shown a high prevalence of *M. hominis* in the vaginal compartment.^23,24^ Based on the high prevalence of this pathogen in the vaginal micro-environment, future studies that investigate its explicit role in this environment are needed.

## References

[1] World Health Organization. Sexually transmitted infections fact sheet. Geneva: World Health Organization; 2019.

[2] Campos GB, Lobao TN, Selis SS, et al. Prevalence of Mycoplasma genitalium and Mycoplasma hominis in urogenital tract of Brazilian women. BMC Infect Dis. 2015;15:60. 10.1186/s12879-015-0792-4 25886914 PMC4336719

[3] Ravel J, Gajer P, Abdo Z, et al. Vaginal microbiome of reproductive – Age women. Proc Natl Acad Sci U S A. 2011;108(Suppl 1):4680–4687. 10.1073/pnas.1002611107 20534435 PMC3063603

[4] Taylor-Robinson D. Mollicutes in vaginal microbiology: Mycoplasma hominis, Ureaplasma urealyticum, Ureaplasma parvum and Mycoplasma genitalium. Res Microbiol. 2017;168(9–10):875–881. 10.1016/j.resmic.2017.02.009 28263902

[5] Redelinghuys MJ, Ehlers MM, Dreyer AW, Lombaard HA, Kock MM. Prevalence of genital mycoplasmas and bacterial vaginosis in pregnant women in Gauteng, South Africa. Sex Transm Infect. 2013;89(Suppl 1):A159. 10.1136/sextrans-2013-051184.0495

[6] Bayraktar MR, Ozerol IH, Gucluer N, Celik O. Prevalence and antibiotic susceptibility of Mycoplasma hominis and Ureaplasma urealyticum in pregnant women. Int J Infect Dis. 2010;14(2):e90–e95. 10.1016/j.ijid.2009.03.020 19515594

[7] Cassell GH, Waites KB, Watson HL, Crouse DT, Harasawa R. Ureaplasma urealyticum intrauterine infection: Role in prematurity and disease in newborns. Clin Microbiol Rev. 1993;6(1):69–87. 10.1128/CMR.6.1.69 8457981 PMC358267

[8] Taylor-Robinson D, Furr PM. Update on sexually transmitted mycoplasmas. Lancet. 1998;351(Suppl 3):12–15. 10.1016/S0140-6736(98)90004-6 9652714

[9] Daxboeck F, Zitta S, Stadler M, Iro E, Krause R. Mycoplasma hominis and Ureaplasma urealyticum in patients with sterile pyuria. J Infect. 2005;51:54–58. 10.1016/j.jinf.2004.06.010 15979492

[10] Patai K, Szilágyi G, Hubay M, Szentmáriay IF, Paulin F. Severe endometritis caused by genital mycoplasmas after Caesarean section. J Med Microbiol. 2005;54:1249–1250. 10.1099/jmm.0.05457-0 16278442

[11] Witt A, Berger A, Gruber CJ, et al. Increased intrauterine frequency of Ureaplasma urealyticum in women with preterm labor and preterm premature rupture of the membranes and subsequent cesarean delivery. Am J Obstet Gynecol. 2005;193(5):1663–1669. 10.1016/j.ajog.2005.03.067 16260207

[12] Pararas MV, Skevaki CL, Kafetzis DA. Preterm birth due to maternal infection: Causative pathogens and modes of prevention. Eur J Clin Microbiol Infect Dis. 2006;25(9):562–569. 10.1007/s10096-006-0190-3 16953371

[13] Arya OP, Tong CY, Hart CA, et al. Is Mycoplasma hominis a vaginal pathogen? Sex Transm Infect. 2001;77(1):58–62. 10.1136/sti.77.1.58 11158693 PMC1758313

[14] Van der Schee C, Sluiters HJ, Van der Meijden WI, et al. Host and pathogen interaction during vaginal infection by Trichomonas vaginalis and Mycoplasma hominis or Ureaplasma urealyticum. J Microbiol Methods. 2001;45(1):61–67. 10.1016/S0167-7012(01)00224-X 11295198

[15] Abbai NS, Wand H, Ramjee G. Sexually transmitted infections in women participating in a biomedical intervention trial in Durban: Prevalence, coinfections, and risk factors. J Sex Transm Dis. 2013;2013:358402. 10.1155/2013/358402 26316957 PMC4436868

[16] Naidoo S, Wand H, Abbai NS, Ramjee G. High prevalence and incidence of sexually transmitted infections among women living in KwaZulu-Natal, South Africa. AIDS Res Therapy. 2014;11(1):1–7. 10.1186/1742-6405-11-31 PMC4168991 25243015

[17] Abbai NS, Wand H, Ramjee G. Socio-demographic and behavioural characteristics associated with HSV-2 sero-prevalence in high risk women in KwaZulu-Natal. BMC Res Notes. 2015;8(1):1–5. 10.1186/s13104-015-1093-0 25940115 PMC4423103

[18] Dessai F, Nyirenda M, Sebitloane M, Abbai N. Diagnostic evaluation of the BD Affirm VPIII assay as a point-of-care test for the diagnosis of bacterial vaginosis, trichomoniasis and candidiasis. Int J STD AIDS. 2020;31(4):303–311. 10.1177/0956462419895684 32050856

[19] Mabaso N, Naicker C, Nyirenda M, Abbai N. Prevalence and risk factors for Trichomonas vaginalis infection in pregnant women in South Africa. Int J STD AIDS. 2020;31(4):351–358. 10.1177/0956462420907758 32075536

[20] Diaz L, Cabrera LE, Fernandez T, et al. Frequency and antimicrobial sensitivity of Ureaplasma urealyticum and Mycoplasma hominis in patients with vaginal discharge. MEDDIC Rev. 2013;15(4):45–47. 10.37757/MR2013V15.N4.11 24253351

[21] Sanchez-Garcia EK, Contreras-Paredes A, Martinez-Abundis E, Garcia-Chan D, Lizano M, De la cruz-Hernandez E. Molecular epidemiology of bacterial vaginosis and its association with genital micro-organisms in asymptomatic women. J Med Microbiol. 2019;68(9):1373–1382. 10.1099/jmm.0.001044 31329097

[22] Panos G. Prevalence studies of M. genitalium and other sexually transmitted pathogens in high risk individuals indicate the need for comprehensive investigation of STIs for accurate diagnosis and effective treatment. GERMS. 2018;8(1):8–11. 10.18683/germs.2018.1127 29564243 PMC5845974

[23] Seifoleslami M, Safari A, Khameneie MK. Prevalence of Ureaplasma urealyticum and Mycoplasma hominis in high vaginal swab samples of infertile females. Iran Red Crescent Med J. 2015;17(12):e16823. 10.5812/ircmj.16823 26756000 PMC4706991

[24] Adebamowo SN, Ma B, Zella D, et al. Mycoplasma hominis and Mycoplasma genitalium in the vaginal microbiota and persistent high-risk human papillomavirus infection. Front Public Health. 2017;5:140. 10.3389/fpubh.2017.00140 28695118 PMC5483445

